# SHH-N non-canonically sustains androgen receptor activity in androgen-independent prostate cancer cells

**DOI:** 10.1038/s41598-021-93971-6

**Published:** 2021-07-21

**Authors:** Diana Trnski, Maja Sabol, Sanja Tomić, Ivan Štefanac, Milanka Mrčela, Vesna Musani, Nikolina Rinčić, Matea Kurtović, Tina Petrić, Sonja Levanat, Petar Ozretić

**Affiliations:** 1 Laboratory for Hereditary Cancer, Division of Molecular Medicine, Ruđer Bošković Institute, Bijenička 54, 10000 Zagreb, Croatia; 2 Laboratory for Protein Biochemistry and Molecular Modelling, Division of Organic Chemistry and Biochemistry, Ruđer Bošković Institute, Bijenička 54, 10000 Zagreb, Croatia; 3 Primary Health Care Center Osijek, Park kralja Petra Krešimira IV 6, 31000 Osijek, Croatia; 4 Faculty of Medicine, Josip Juraj Strossmayer University of Osijek, Josipa Huttlera 4, 31000 Osijek, Croatia; 5 Department of Pathology, Clinical Hospital Centre Osijek, Josipa Huttlera 4, 31000 Osijek, Croatia

**Keywords:** Molecular medicine, Oncology, Oncogenes, Urological cancer

## Abstract

Prostate cancer is the second most frequent cancer diagnosed in men worldwide. Localized disease can be successfully treated, but advanced cases are more problematic. After initial effectiveness of androgen deprivation therapy, resistance quickly occurs. Therefore, we aimed to investigate the role of Hedgehog-GLI (HH-GLI) signaling in sustaining androgen-independent growth of prostate cancer cells. We found various modes of HH-GLI signaling activation in prostate cancer cells depending on androgen availability. When androgen was not deprived, we found evidence of non-canonical SMO signaling through the SRC kinase. After short-term androgen deprivation canonical HH-GLI signaling was activated, but we found little evidence of canonical HH-GLI signaling activity in androgen-independent prostate cancer cells. We show that in androgen-independent cells the pathway ligand, SHH-N, non-canonically binds to the androgen receptor through its cholesterol modification. Inhibition of this interaction leads to androgen receptor signaling downregulation. This implies that SHH-N activates the androgen receptor and sustains androgen-independence. Targeting this interaction might prove to be a valuable strategy for advanced prostate cancer treatment. Also, other non-canonical aspects of this signaling pathway should be investigated in more detail and considered when developing potential therapies.

## Introduction

Prostate cancer is the second most frequent cancer in men worldwide^1^. With the aid of prostate-specific antigen (PSA) screening most prostate cancer cases are diagnosed and treated while the disease is still localized. However, there are still many patients that develop high-risk localized, locally advanced, or metastatic cancer^2^. Since androgen receptor (AR) signaling is the main pathway ensuring prostate cancer cell growth, androgen deprivation therapy (ADT) has been the standard treatment option for patients with advanced forms of prostate cancer. Even though this therapy is effective initially, most patients progress to a poor-prognosis stage called castration-resistant prostate cancer (CRPC). A major characteristic of this stage is that despite the low levels of androgen, AR signaling continues to be activated through alternative mechanisms and promotes the growth of CRPC^3^. Several AR-dependent and independent mechanisms leading to CRPC have been proposed. AR-dependent mechanisms include AR mutation or overexpression, expression of constitutively active AR splice variants, altered expression of AR co-regulators to non-canonical AR transactivation, all leading to increased AR activity^4^. Several AR-independent mechanisms involving signaling pathways able to bypass the AR, or the existence of AR-negative prostate cancer stem cells, ultimately leading to the development of CRPC^5^ have also been identified. To date none of these mechanisms can completely account for the progression to CRPC. Apparently, additional research is needed to elucidate the specificities of these mechanisms that could lead to the development of more effective treatment.

The Hedgehog-GLI (HH-GLI) signaling pathway is involved in many aspects of embryonic development as well as stem cell maintenance and tissue homeostasis in adult organisms^6^. Aberrant activation in adult cells has been linked with the development of various tumors, including prostate cancer^7–11^. In mammals, signaling is initiated by the 50 kDa Hedgehog (HH) protein which serves as the pathway ligand. Mammals express three different Hedgehog ligands (Sonic, Desert and Indian Hedgehog; SHH, DHH and IHH, respectively) consisting of an N-terminal signaling domain and a C-terminal autoprocessing domain. To become a fully active signaling molecule (HH-N), the C-terminal domain autocatalytically initiates cleavage of the protein and addition of a cholesterol moiety to the signaling domain’s C-terminus. Additionally, a palmitoyl group is added to the N-terminus of the signaling domain. Dual lipidation of the HH signaling peptide is essential both for its activity and the regulation of its spread through tissues^12,13^.

Binding of any of the Hedgehog ligands to the transmembrane receptor Patched1 (PTCH1) initiates the canonical pathway by releasing the co-receptor Smoothened (SMO), otherwise suppressed by PTCH1. A rapid translocation of SMO to the primary cilium follows, leading to the dissociation of the GLI transcription factors from their regulator Suppressor of Fused (SUFU). The GLI proteins translocate to the nucleus and promote target gene transcription. Mammals have three different GLI transcription factors: GLI1, GLI2 and GLI3. GLI2 and GLI3 harbor a N-terminal repressor domain and can act as both activators and repressors of the pathway, while GLI1, lacking this domain, acts only as a transcriptional activator. In absence of the HH signal, the GLI proteins remain bound to SUFU which enables their phosphorylation and subsequent degradation in the proteasome, leading to the formation of GLI2 and GLI3 repressors which block target gene activation^14,15^.

In recent years it has become evident that apart from the canonical Hedgehog signaling cascade, non-canonical signaling branches through PTCH1 or SMO exist^16^. It has been proposed that SMO can signal through G proteins leading to activation of downstream molecules such as SRC Family Kinases^17^. Also, activation of the GLI transcription factors by other signaling pathways can ensure pathway activity in many cancer types, independently of the upstream signaling cascade^18^. Interestingly, in prostate cancer cells a direct interaction between the AR and the GLI proteins has been found. This interaction is proposed to lead to activation of both AR and HH-GLI pathways, and subsequently to cell proliferation^19,20^.

We studied upstream HH-GLI signaling and its role in AR signaling and androgen resistance. We identified a potential new mechanism of AR activation by the HH-GLI ligand SHH-N in androgen-independent cells.

## Results

### HH-GLI pathway components are expressed in LNCaP cells

**Figure 1:**
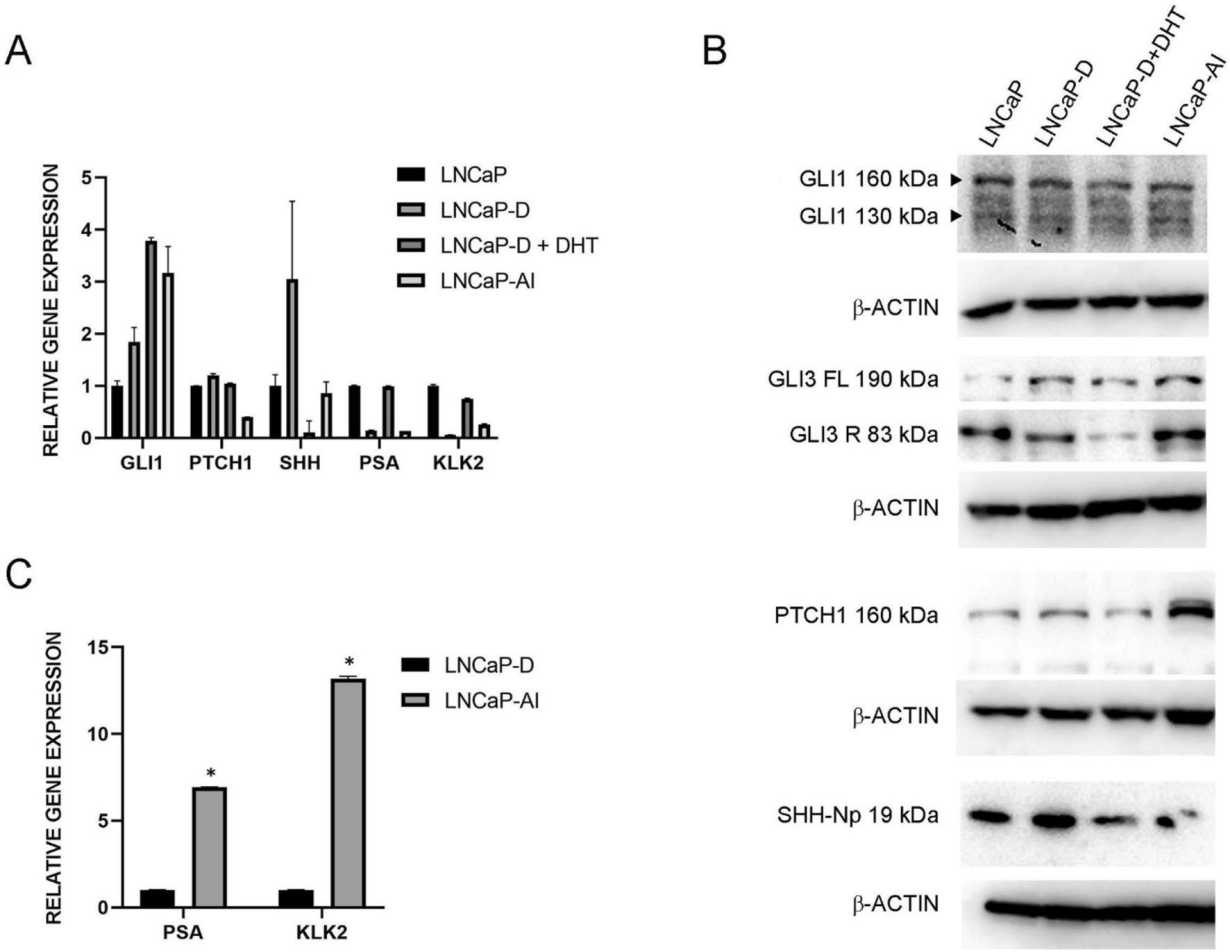
Differences in HH-GLI and AR pathways among the LNCaP cell line variants (LNCaP, LNCaP-D, LNCaP-D + DHT and LNCaP-AI). (**A**) The HH-GLI and AR target genes expression levels. *SHH*, *PSA* and *KLK2* are androgen regulated genes with their expression depending on androgen levels. (**B**) Protein expression levels of key components of the HH-GLI signaling pathway. (**C**) AR target gene expression levels in androgen-independent LNCaP-AI cells are much higher than in cells exposed to short-term androgen deprivation (LNCaP-D). *Indicates P < 0.05.

To verify the androgen independence of the generated LNCaP-AI cells we calculated and compared the growth rate of the three LNCaP cell variants in androgen depleted medium. Androgen independent cells have a higher growth rate in depleted conditions (1.76), which was shown for our LNCaP-AI cell line when compared to wild type LNCaP cells (0.59) or short-term depleted cells LNCaP-D (0.43). Exogenously added DHT also had no significant effect on LNCaP-AI proliferation, as it did on wild type and LNCaP-D proliferation (Figure S1, Table S1). LNCaP cells and their variants LNCaP-D, LNCaP-D + DHT and LNCaP-AI express *GLI1*, *PTCH1*, *SHH* as well as the AR target genes *PSA* and *KLK2*. The highest variability in gene expression levels among the cell lines was detected for *SHH*, *PSA* and *KLK2*, all androgen regulated genes (Fig. 1A). Expression of the androgen repressed gene *SHH* increased after short-term androgen depletion in LNCaP-D cells, while it was downregulated after addition of DHT, which could also be seen on protein levels (Fig. 1B). On the other hand, long term androgen depletion and androgen independence did not have any effect on *SHH* levels. *PSA* and *KLK2* are direct AR targets and are downregulated after short-term androgen depletion, but their expression is restored after DHT addition. The full length GLI1 protein did not reflect the changes in *GLI1* gene expression. GLI3 protein processing was inhibited after addition of DHT, which supports the finding of Li et al. that in the presence of androgen AR binds GLI3 and inhibits its processing into the repressor form^20^. PTCH1 protein, a negative regulator of HH-GLI signaling, was upregulated after long term androgen deprivation. *PTCH1* gene expression levels on the other hand, were downregulated under these conditions, suggesting inhibition of the canonical HH-GLI pathway (Fig. 1A,B). In LNCaP-AI cells *PSA* and *KLK2* expression was lower compared with LNCaP cells but elevated in comparison with their levels after short-term androgen deprivation, suggesting that AR activity was partly restored by alternative mechanisms to sustain androgen-independent growth (Fig. 1C).

### Canonical and non-canonical HH-GLI signaling

**Figure 2:**
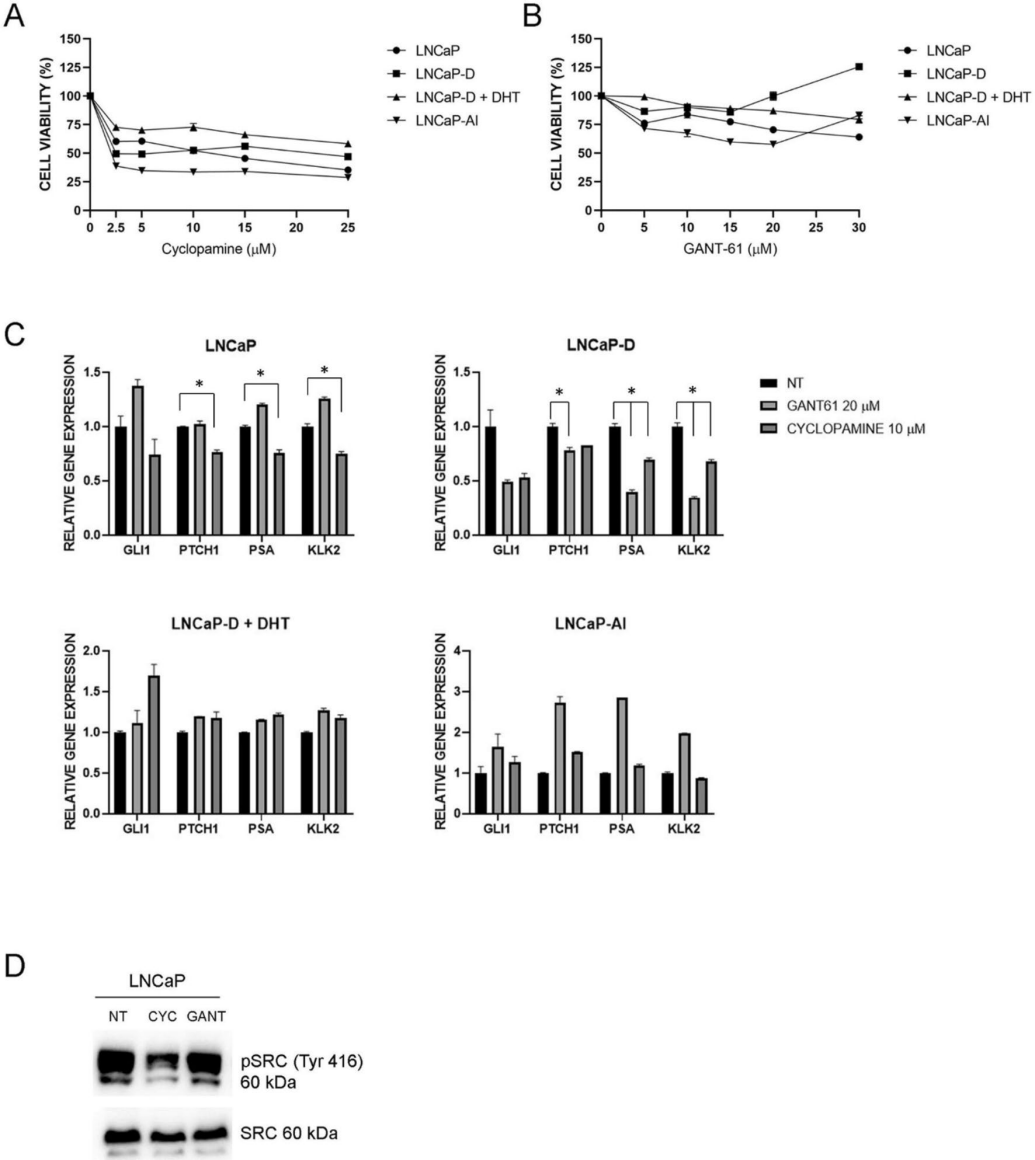
Effects of HH-GLI signaling inhibitors on cell proliferation and pathway activity. (**A**) Effects of cyclopamine on proliferation of LNCaP, LNCaP-D, LNCaP-D + DHT and LNCaP-AI cells. Cyclopamine elicits an inhibitory effect on cell proliferation. (**B**) Effects of GANT-61 on proliferation of LNCaP, LNCaP-D, LNCaP-D + DHT and LNCaP-AI cells. GANT-61 elicits only a weak effect on cell proliferation. (**C**) Effects of cyclopamine and GANT-61 on HH-GLI and AR pathway activity. Cyclopamine partly downregulates HH-GLI signaling and downregulates AR signaling in LNCaP cells. Both inhibitors show an inhibitory effect on HH-GLI and AR pathway activity after short-term androgen deprivation (LNCaP-D). (**D**) In LNCaP cells, cyclopamine inhibits SRC activation, measured by its phosphorylation at Tyr416, while the levels of total SRC remain constant. Significant downregulation (P < 0.05) is indicated with *.

After establishing the cell lines, we tested how inhibition of the HH-GLI signaling pathway affects their growth. We treated the cells with cyclopamine, a SMO inhibitor, and GANT-61, a direct GLI inhibitor. Interestingly, cyclopamine did inhibit cell proliferation, but the effect of GANT-61 was very weak (Fig. 2A,B). We checked the effect of these compounds on HH-GLI signaling activity, as well as AR signaling activity. Cyclopamine slightly downregulated *PTCH1* expression in LNCaP cells and downregulated AR target genes, whereas GANT-61 had no effect on these pathways. In LNCaP-D cells there seemed to be a more pronounced effect of both compounds on HH-GLI pathway activity, but only *PTCH1* downregulation by GANT-61 was statistically significant. On the other hand, both compounds significantly downregulated AR signaling. No effect of either cyclopamine or GANT-61 was seen in LNCaP-D + DHT or LNCaP-AI cells (Fig. 2C). This suggests that short-term androgen deprivation activates HH-GLI signaling.

Since cyclopamine showed only a mild effect on HH-GLI and AR signaling but downregulated LNCaP cell proliferation we checked whether non-canonical SMO signaling through SRC could be active alongside the canonical pathway. In LNCaP cells we observed elevated levels of active SRC protein phosphorylated at Tyr416, pSRC (Tyr416). Cyclopamine addition inhibited this phosphorylation, resulting in a pSRC (Tyr416) reduction, suggesting that SMO non-canonically activates SRC in wild type LNCaP cells (Fig. 2D). However, LNCaP cells exposed to either short- or long-term androgen deprivation (LNCaP-D and LNCaP-AI) expressed only low levels of activated pSRC (Tyr416) and we did not observe this effect (data not shown).

### Activation of AR by SHH in androgen-independent cells

**Figure 3:**
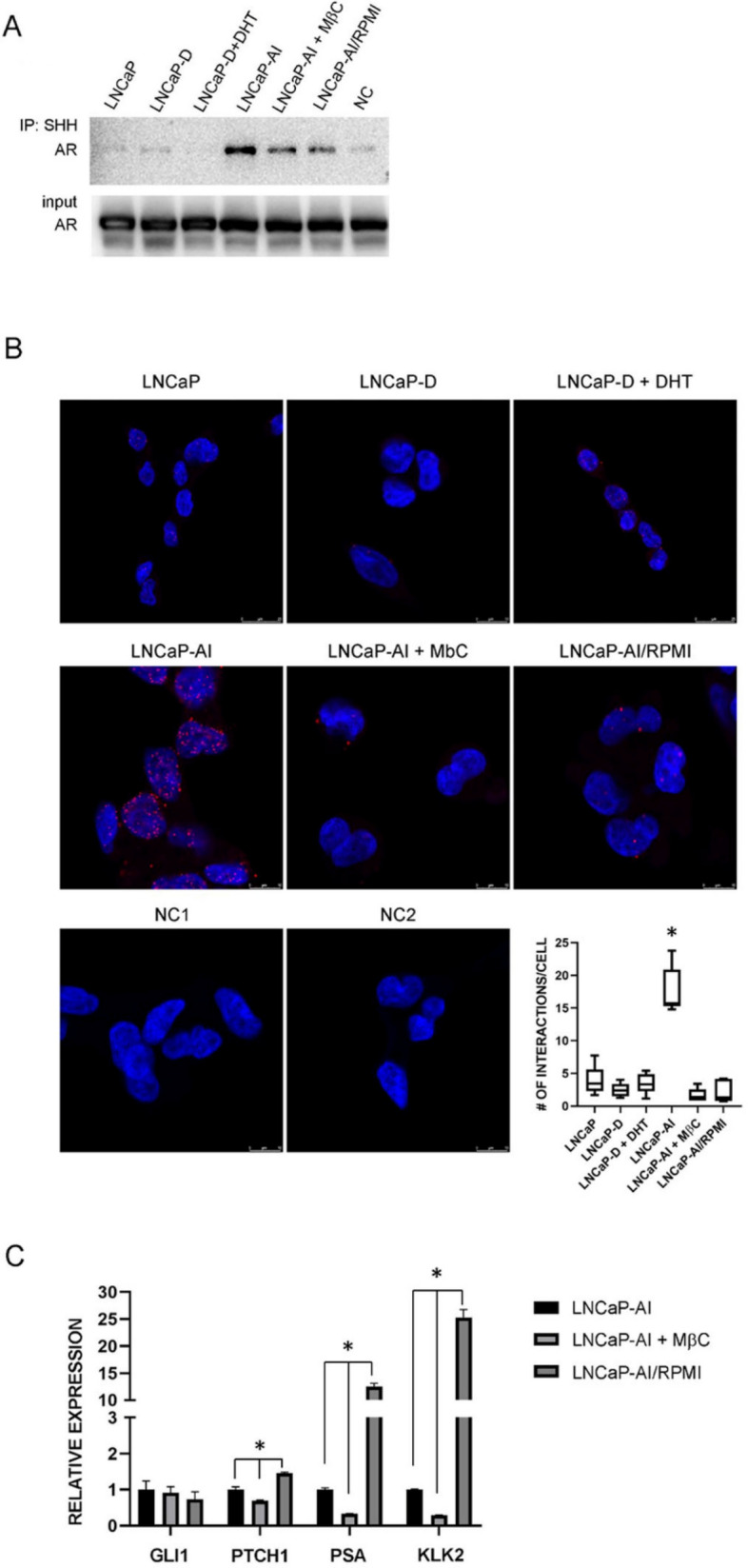
Evidence of SHH-N and AR direct interactions. (**A**) Co-immunoprecipitation analysis showed a direct SHH-N–AR interaction only in androgen-independent cells LNCaP-AI. The interaction was reduced after cholesterol depletion with MβC or by growing the cells in medium with non-CS-FBS (RPMI). (**B**) Proximity ligation assay confirmed this interaction, depicted by the highest number of red dots per cell in LNCaP-AI cells, also represented graphically, N = 6 for LNCaP cells and N = 5 for LNCaP-D, LNCaP-D + DHT, LNCaP-AI, LNCaP-AI + MbC and LNCaP-AI/RPMI. Nuclei are shown in blue, points of interaction are shown in red, NC—negative control. (**C**) Cholesterol depletion downregulated AR signaling activity, suggesting that the SHH-N–AR interaction sustains AR activity in androgen-independent cells. Growing the cells in medium with non-CS-FBS upregulated AR signaling, confirming higher affinity of AR for androgen. *Indicates P < 0.05.

Since we found little evidence of canonical HH-GLI activation in our cell lines, driven by our previous findings in breast cancer cells^21^ where we found evidence of SHH and ERα interactions, we checked whether SHH-N could non-canonically bind the AR and contribute to its activity. We hypothesized that SHH-N could bind the AR through its cholesterol modification, since androgen is synthesized from cholesterol and they are structurally similar. Interestingly, we confirmed, by both co-immunoprecipitation and PLA, that a complex between SHH-N and AR forms, but only in androgen-independent LNCaP-AI cells. After cholesterol depletion with methyl-β-cyclodextrin this complex formation was reduced, indicating the interaction is cholesterol dependent. Also, when the androgen-independent cells were returned to non-CS-FBS RPMI medium, this interaction was inhibited, suggesting the affinity of AR for SHH-N is lower than its affinity for androgens (Fig. 3A,B).

To confirm that the binding of SHH-N to AR activated AR signaling, we tested whether cholesterol depletion or growing the cells in non-CS-FBS RPMI medium, which inhibited this interaction, had any effect on HH-GLI or AR signaling activity. We showed that inhibiting the SHH-N–AR complex formation by cholesterol depletion decreased AR target gene expression, suggesting that SHH-N activates the AR in androgen-independent cells. On the other hand, this treatment had only slightly affected the HH-GLI activity, as indicated by a slight *PTCH1* downregulation, implying a redundant role for the canonical pathway in androgen-independent cells. Upregulation of AR target genes in LNCaP-AI cells growing in non-CS-FBS RPMI medium, suggests that androgen, as the primary AR ligand, is much more potent in activating the AR than SHH-N. Also, growing LNCaP-AI cells under these conditions slightly upregulated *PTCH1* expression (Fig. 3C). To confirm a role for SHH in LNCaP-AI cells we silenced *SHH* which led to a downregulation of AR target genes *PSA* and *KLK2* in these cells (Figure S2A,B). Interestingly, when LNCaP-AI cells were grown as spheroids, a more biologically relevant model of tumor growth, *SHH* expression increased while PTCH1 and GLI1 remained unchanged (see SI and Figure S2C,D). Consequently, an increase in *PSA* and *KLK2* expression was observed in LNCaP-AI spheroids (Figure S2D). Together, this data suggests that SHH impacts AR activity in LNCaP-AI cells.

### Molecular modelling of the SHH-N and AR complex

**Figure 4:**
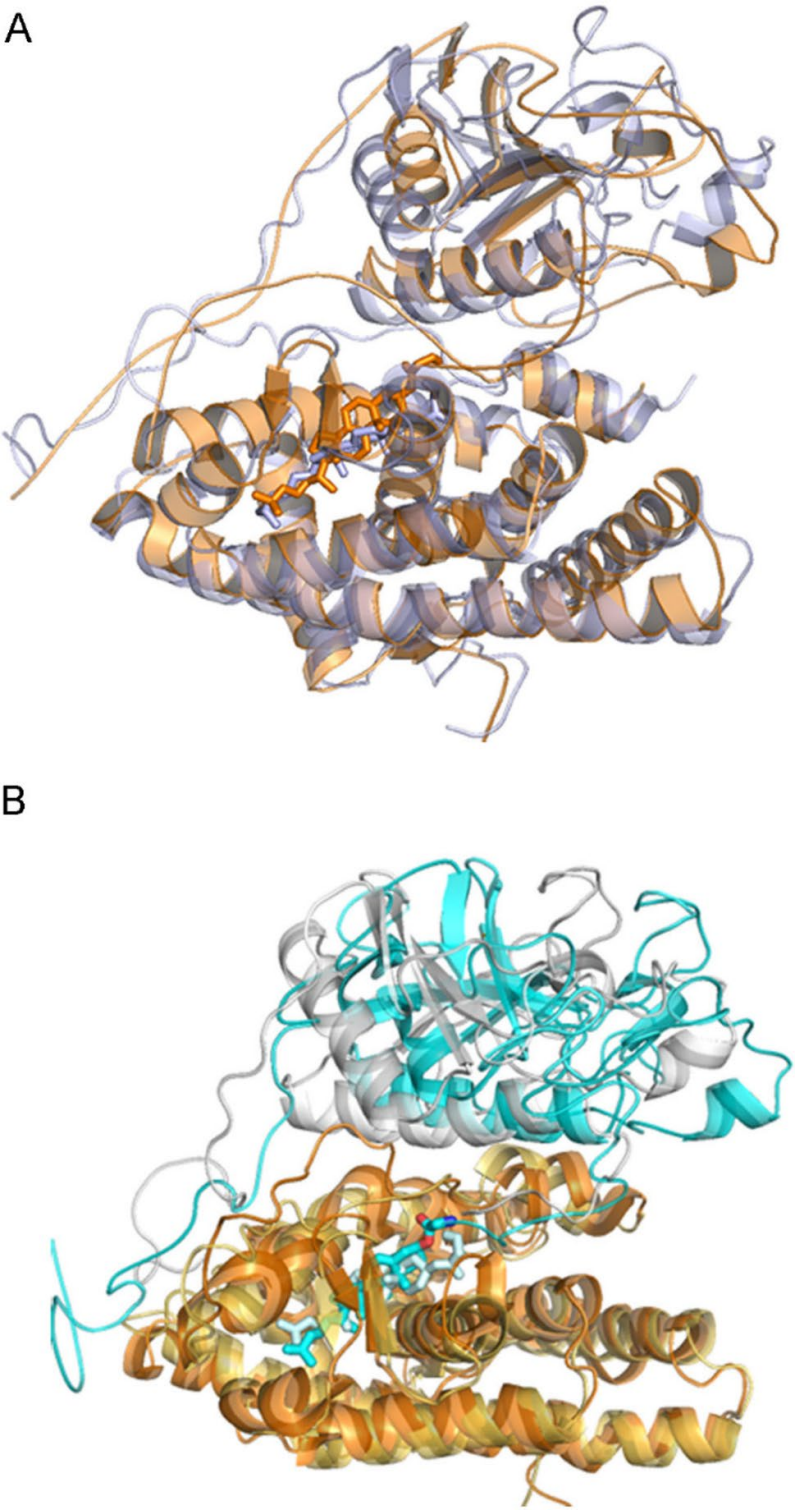
Molecular modelling of the ARwt–SHH-N–cholesterol complexes. (**A**) Overlay of the ARwt–SHH-N–cholesterol structures: docked (gray) and at the end of 100 ns of MD simulation (orange). (**B**) Orientation of the cholesterol SHH-N molecule in the AR–SHH-N–cholesterol complexes at the end of 100 ns of MD simulation: in the complex with the wild type, ARwt (cyan) and in the complex with its T878A mutant (light blue). Alignment was performed on AR. Cholesterol molecules in the aligned complexes are represented as sticks.

To investigate the possibilities of SHH-N to AR binding, as well as stability of the formed complexes, we performed MD simulation study. Complexes between AR (both, wild type and its T878A homologue) and cholesteroylated N-terminal domain of SHH were built (Figure S3) and for each, ARwt–SHH-N–cholesterol and T878A–SHH-N–cholesterol complex, two, 100 ns long MD simulations were performed. Complexes remained stable and conformations of the interactors remained mostly conserved during MD simulations (Figure S4) but their mutual orientation changed (Fig. 4A). Also, although the ligand binding pocket in AR is very tight and there is not much freedom for cholesterol to reorient, slight orientations of the ligand were noticed during MD simulations, especially in the T878A–SHH-N–cholesterol complex (Fig. 4B and Figure S4). Namely, in the ARwt–SHH-N–cholesterol complex Thr878 stabilizes cholesterol through weak electrostatic, O⋯H, and van der Waals (vdw) stabilization which are in the T878A–SHH-N–cholesterol complex replaced with just a very weak vdw stabilization with Ala.

**Figure 5:**
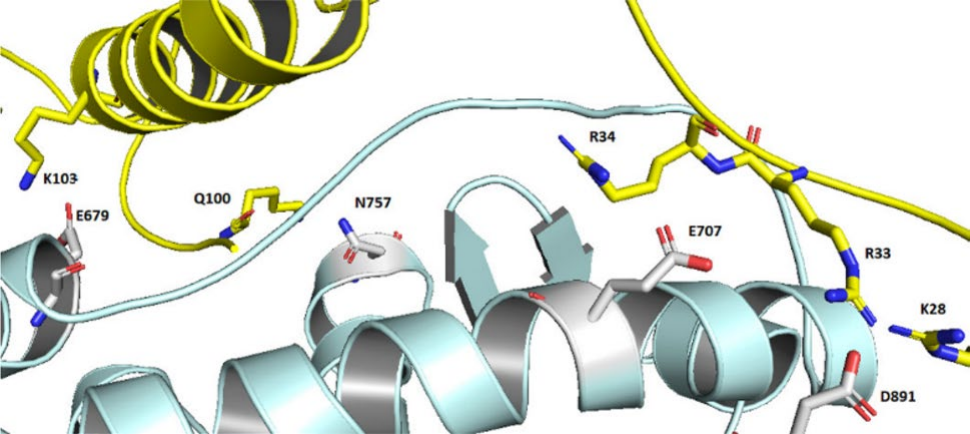
Amino acid residues that form the strongest intermolecular AR (pale blue)—SHH-N (yellow) interactions during MD simulations.

MMGBSA approximation of the AR to SHH-N–cholesterol binding free energy was calculated (for details see “Materials and methods”) for 20 ns and 40 ns long segments (Table S2) sampled during MD simulations, and analysis of the intermolecular, protein–protein and protein–ligand, hydrogen bonds was performed for each trajectory. According to the calculated MMGBSA energies it seems that the complex between N-terminal domain of SHH with covalently bound cholesterol, binds to the wild type AR with slightly higher affinity than to its T878A homologue (< ΔE_MMGB_ > _100 ns_ values are − 96 kcal/mol and − 84 kcal/mol, respectively). Such result is in agreement with the population of the intermolecular, protein–protein hydrogen bonds (Table 1) which is higher in AR–SHH-N–cholesterol than in T878A–SHH-N–cholesterol complex. The most populated intermolecular hydrogen bonds are (AR–SHH-N): Asp891-Arg28/Arg33, Glu679-Q100/K103/K194, and Glu707-R34 (Fig. 5, Table S3), wherein AR participates in the interactions mostly with negatively (Glu and Asp) and SHH-N mostly with positively charged amino acids (Arg and Lys).

**Table 1:** Population of the intermolecular hydrogen bonds.

	< ΔE_MM-GB_ > _100 ns_ (kcal/mol)	Hydrogen bonds population (%)
WT-1	− 96 (± 15)	984
WT-2	− 84 (± 11)	868
MUT-1	− 84 (± 14)	835
MUT-2	− 79 (± 12)	605

The analysis was performed for the each 100 ns long trajectory (on 20–40 ns long segments, see Table S3). The hydrogen bonds population is given as a sum of all intermolecular protein–protein hydrogen bonds sampled during 100 ns long MD trajectories. The hydrogen bonds occurring < 3% in all of the sampled structures are omitted from the sum.

*WT* wild type AR, *MUT* mutated AR (T878A).

The hydrogen bonds population between residues (i and j) is calculated as the ratio of the number of trajectory frames containing hydrogen bond and the total number of frames. (HBi,jpop = N(frames with HBi,j)/N(frames total)). In the case of residues forming multiple hydrogen bonds, a sum of these values is given, which allows values larger than 100%.

## Discussion

The contribution of the HH-GLI signaling pathway to prostate cancer development as well as interactions of the HH-GLI and AR signaling pathways have been intensely investigated over the past two decades. Early research suggested autocrine HH-GLI signaling is key for sustaining prostate cancer growth^22–24^. However, this model was later reconsidered as it became evident that activation of SMO in a mouse model did not lead to neoplastic transformation of the prostate as it did of skin, brain and muscle tissue^25^. Also, Zhang et al. found no evidence of autocrine HH-GLI signaling in three prostate cancer cell lines, LNCaP, PC3 and 22RV1. They also found that cyclopamine inhibited cell proliferation but did not affect canonical HH-GLI pathway activity^26^. On the other hand, research on xenografts revealed paracrine HH-GLI signaling is more likely to support prostate cancer cell growth^27,28^. As *SHH* is an androgen repressed gene, it was found that prolonged exposure of LNCaP cells to androgen deprived conditions enables limited activation of autocrine HH-GLI signaling in response to HH ligands that are produced under these conditions, proposing a role of HH-GLI signaling in sustaining androgen-independent growth of prostate cancer cells^9,29^. Later on, a direct interaction between the HH-GLI and AR signaling pathways was found, suggesting a completely new, non-canonical function for the HH-GLI signaling pathway in prostate cancer. GLI2 binds the AR and this might be the basis for HH-GLI support of AR activity under androgen-deprived conditions^19^. Recently, this group discovered that GLI3 also forms a complex with the liganded AR. This prevents GLI3 processing and stabilizes it in its active conformation leading to upregulated transcription of GLI target genes^20^.

In this paper we aimed to investigate the role of HH-GLI signaling in androgen-independent growth of prostate cancer cells in more detail. After establishing the androgen independent cell line LNCaP-AI we observed an upregulation in PTCH1 protein expression, as well as upregulated *GLI1* mRNA expression, that have also been found in clinical CRPC samples by Shaw et al.^9^. In addition to its increased growth rate in androgen deprived medium, these findings support the androgen independence of the LNCaP-AI cell line. Similar to previous findings, we showed that LNCaP cell proliferation is inhibited with cyclopamine, but canonical HH-GLI was only slightly affected. Only in LNCaP-D cells both HH-GLI and AR signaling pathways were downregulated and this effect was reversed by DHT, indicating canonical pathway activation under androgen deprived conditions. Similar results were obtained for GANT-61, but the effect on cell proliferation was minor. On the other hand, the HH-GLI and AR signaling pathways were not affected by cyclopamine or GANT-61 in androgen-independent LNCaP-AI cells. Interestingly, in LNCaP cells we observed that SMO signals non-canonically through the SRC kinase, as pSRC (Tyr416) levels were reduced after cyclopamine treatment. Also, cyclopamine downregulated AR signaling in these cells. It has previously been shown that SMO, as a GPCR like protein, can non-canonically signal through G-proteins that activate the SRC Family Kinases^30,31^. Since it is known that the SRC kinase promotes AR transactivation function^32,33^ this could be the mechanism through which upstream HH-GLI signaling sustains AR signaling and contributes to prostate cancer cell growth. The lack of canonical HH-GLI signaling in these cells and our previous findings of potential SHH-N and ERα interactions^21^, motivated us to investigate whether SHH-N can non-canonically bind and activate the AR in prostate cancer cells. Indeed, we showed that SHH-N binds the AR, but only in androgen-independent LNCaP-AI cells. This is a cholesterol-dependent interaction since depletion of cholesterol decreased complex formation. Molecular modelling study revealed molecular details of this interaction. It showed that SHH-N is able to bind both, the wild-type AR and the mutated T878A form expressed in LNCaP cells. The cholesterol molecule attached to the SHH-N C-terminal tightly binds into the ligand binding site of AR while the two proteins pair to each other by their complementary polar regions forming stable complexes. MMGBSA energies suggest that SHH-N binds to the wild-type AR with slightly higher affinity than to the T878A mutant. This could mean that in androgen-independent cells harboring wild type AR this interaction could be even more prominent. This newly identified interaction between SHH-N and AR is one potential mechanism of sustaining androgen-independent growth of prostate cancer cells and could represent a new therapeutic target. Our results suggest this interaction is specific to androgen-independent cells as they grow in conditions where no androgen is present and need alternative modes of AR activation for survival. So, under these conditions AR cannot be activated by androgen, but SHH-N could take its place through its cholesterol moiety. An upregulation of SHH-N and DHH-N expression in the epithelial compartment of hormone-treated and hormone-refractory prostate cancer samples was associated with the highest risk of biological recurrence^34^, which supports the role of the Hedgehog ligand as a key player in androgen-independent growth. Since cholesterol is the key of SHH-N binding to the AR, a possible means of targeting this interaction could involve cholesterol depletion. Statins, that have been widely used in the clinic as cholesterol-lowering drugs, could potentially have an effect in this case. Epidemiological studies reported a reduced risk of advanced prostate cancer in patients who were taking statins, but no significant results were observed on overall incidence of prostate cancer^35^. This study is in concordance with our finding where SHH-N supports only androgen-independent cell growth and therefore this therapy could prove efficient only for CRPC that relies on the SHH-N–AR interaction, or in combination with ADT for advanced prostate cancer to prevent the interaction from occurring and enabling androgen-independent growth. However, a recent study reported pro-tumorigenic effects of low-dose statins in prostate cancer^36^ and therefore caution is advised and more detailed research of the effect of statins on different stages of prostate cancer should be conducted.

In conclusion, we would like to emphasize the importance of HH-GLI signaling in prostate cancer. Even though it might seem its role is contradictory, deeper insights reveal that its role may not be as straightforward as in other types of cancer. It is becoming more and more evident that canonical HH-GLI signaling might not be the leading cause of prostate cancer development, rather that it is essential for survival of prostate cancer cells under androgen deprived conditions. Also, our findings and those of other groups, highlight the ability of certain HH-GLI components to non-canonically interact with the AR and activate it, which present mechanisms of sustaining androgen-independent growth. As for the SHH-N–AR interaction that we identified, the extent of this interaction in clinical samples should be evaluated and it remains to be investigated whether its inhibition in advanced prostate cancer and CRPC could be relevant for successful treatment.

## Materials and methods

### Cell culture

In this study the prostate cancer cell line LNCaP, obtained from the ATCC (a kind gift from Maja Herak Bosnar, PhD) was used, which is known to express the mutated AR (p.T878A)^37^. LNCaP cells (passages < 40) were maintained in RPMI-1640 medium supplemented with 10% fetal bovine serum (FBS, Sigma), 2 mM l-glutamine and 1000 U/mL penicillin/streptomycin. To investigate HH-GLI signaling in androgen-independent growth, LNCaP cells were cultured in RPMI-1640 supplemented with charcoal-stripped FBS (CS-FBS), l-glutamine and penicillin/streptomycin for 5 days (LNCaP-D). To check if the observed effects after short term androgen deprivation are androgen specific, 5 nM dihydrotestosterone (DHT, SelleckChem) was added to the medium (LNCaP-D + DHT). Androgen-independent cells (LNCaP-AI) were generated by growing LNCaP cells in RPMI-1640 supplemented with charcoal-stripped FBS (CS-FBS), l-glutamine and penicillin/streptomycin > 10 months. To confirm that LNCaP-AI cells have indeed become independent of androgen, growth rates of cell lines in androgen depleted conditions were calculated as the growth for 5 days in depleted medium/growth in complete medium^9^.

For gene and protein expression experiments, 24 h after seeding cells were treated with 10 µM SMO inhibitor cyclopamine (Toronto Research Chemicals) or 20 µM GLI inhibitor GANT-61 (SelleckChem) for 48 h. For cholesterol depletion, LNCaP-AI cells were treated with 1 mM methyl-β-cyclodextrin (Sigma) for 24 h. All experiments were performed at least in duplicate.

### Cell proliferation assay

Cell proliferation and viability were determined using MTT assay as previously described^38^. The cells were treated in quadruplicates for 72 h with 0.5–5 µM cyclopamine or 5–30 µM GANT-61. All experiments were performed in triplicate.

### Quantitative real-time PCR (qRT-PCR)

RNA from cells was extracted using Nucleozol reagent (Macherey–Nagel) according to manufacturer’s instructions. 1 µg of RNA was reverse transcribed into cDNA using the High-Capacity cDNA Reverse Transcription Kit (Thermo Fisher). Gene expression was analyzed on the CFX96 real-time PCR machine (Bio-Rad) using the SsoAdvanced Universal SYBR Green Supermix (Bio-Rad) and the primers listed in Table S4. All experiments were performed in triplicate.

### Western blot

Proteins were extracted using RIPA buffer supplemented with protease inhibitors (Roche). For analysis of phosphorylated proteins phosphatase inhibitors (Roche) were also added as described previously^38^. Protein concentration was determined using the Pierce BCA Protein Assay Kit (Thermo Fisher). Primary antibodies against AR (#5153, Cell Signaling Technology, 1:1000), SHH (sc-365112, Santa Cruz Biotechnology, 1:100), PTCH1 (A0826, ABclonal, 1:2000), GLI1 (#3538, Cell Signaling Technology, 1:200), GLI3 (GTX104362, GeneTex, 1:1000), SRC (#2123, Cell Signaling Technology, 1:1000) and pSRC (Tyr416) (#6943, Cell Signaling Technology, 1:1000) were used. β-ACTIN (60008-1-Ig, ProteinTech, 1:4000) was used as loading control.

### Co-immunoprecipitation

For co-immunoprecipitation experiments Protein G coated Dynabeads (Thermo Fisher) were used according to manufacturer’s instructions as previously described^38^. For SHH co-IP 5 µg of SHH antibody (sc-365112, Santa Cruz Biotechnology) was used per sample. An irrelevant IgG antibody, known not to have any interactions with the AR, was used as negative control.

### Proximity ligation assay (PLA)

For PLA cells were seeded on Milli EZSlide 8-well glass slides (Merck). 24 h after seeding the cells were fixed with 4% paraformaldehyde and permeabilized with methanol. Epitope retrieval was performed using sodium citrate buffer, pH 6.0. Blocking and further steps of the assay were performed using Duolink PLA Technology (Sigma Aldrich) reagents as per manufacturer’s instructions: Duolink PLA Probes anti-rabbit PLUS (DUO92002), anti-mouse MINUS (DUO92004), Duolink detection agent Red (DUO92008), Duolink wash buffers (DUO82049-4L). The samples were incubated with the primary antibodies against SHH (sc-365112, Santa Cruz Biotechnology, 1:50) and AR (#5153, Cell Signaling Technology, 1:400) in a humidity chamber at + 4 °C overnight. LNCaP-AI cells were also used for negative controls: in one negative control no primary antibodies were used, as for the second, instead of the anti-SHH antibody, anti-γ-tubulin was used that should not interact with the AR. The next day the cells were mounted in Prolong Gold Antifade mounting medium with DAPI (Thermo Fisher). Confocal images were obtained with the Leica SP8 X FLIM confocal microscope (Leica Microsystems). The cell nuclei are stained blue with the DAPI stain, whereas the points of SHH-N and AR interactions are shown as red dots in the cells.

### Molecular modelling

The AR-SHH-cholesterol complex was built using structures available in PDB^39^: the X-ray structure of human AR ligand binding domain in complex with dihydrotestosterone, PDB code 2AM9 (chain A, resolution 1.64 Å) and the C-terminally cholesteroylated N-terminal domain of SHH determined by cryo electron microscope (PDB code 6RVD, chain C, resolution 3.5 Å).

The complexes between both the wild type AR and its T878A mutant were built (for details see Supplementary Information (SI)) and simulated in explicit, TIP3P type^40^ water. The obtained complexes were parametrized by the AmberTools16 module tleap using ff14SB force field^41^. Cholesterol was parametrized within the gaff force field^42^ and AM1-BCC charges were assigned. For the zinc cation, nonbonded parameters developed by us were used^43^. For each complex two independent 100 ns long molecular dynamic (MD) simulations were performed (details of the systems preparation and MD simulations, as well as the data analysis are given in SI). Finally, the AR-SHH-cholesterol binding free energies were calculated using single trajectory MMGBSA approach on 20 ns and 40 ns long intervals sampled throughout the trajectory sampled during 100 ns of MD simulation. The calculations were performed by MMPBSA.py script available within the Amber software suite version 16 (https://ambermd.org/)^44^.

### Statistical analysis

D'Agostino-Pearson test was used for testing normality of data distribution. Non-normal data were log transformed. Independent samples t-test and one-way ANOVA with Dunnett's post-hoc test were used for inferring the differences in genes’ expression. One-way ANOVA with Student–Newman–Keuls post-hoc test was used for analyzing PLA results. Two-tailed P-values < 0.05 were considered statistically significant. Statistical analyses were performed with MedCalc v19.2.1 (MedCalc Software Ltd) and Prism 8 (GraphPad Software).

## Supplementary Information

Supplementary Information.

## Data Availability

All data generated or analyzed during this study are included in this article and its supplementary information file. Any other details are available from the corresponding authors on reasonable request.
