# Supplementary material for: SHH-N non-canonically sustains androgen receptor activity in androgen-independent prostate cancer cells
Source: Sci Rep. 2021 Jul 21;11:14880. doi: 10.1038/s41598-021-93971-6 (PMC8295376; doi:10.1038/s41598-021-93971-6)

**SUPPLEMENTARY INFORMATION**

**SHH-N non-canonically sustains androgen receptor activity in androgen-independent LNCaP prostate cancer cells**

**Authors:** Diana Trnski*, Maja Sabol, Sanja Tomić*, Ivan Štefanac, Milanka Mrčela, Vesna Musani, Nikolina Rinčić, Matea Kurtović, Tina Petrić, Sonja Levanat, Petar Ozretić

***Corresponding authors:** *Diana Trnski*, Laboratory for Hereditary Cancer, Division of Molecular Medicine, Ruđer Bošković Institute, Bijenička 54, Zagreb, 10000, Croatia. Tel.+385-1-4571292, Fax: +385-1-4561010, e-mail: Diana.Trnski@irb.hr; *Sanja Tomić*, Laboratory for Protein Biochemistry and Molecular Modelling, Division of Organic Chemistry and Biochemistry, Ruđer Bošković Institute, Bijenička 54, Zagreb, 10000, Croatia. Tel. +385-1-4571251, e-mail: Sanja.Tomic@irb.hr

**Supplementary Figures S1-S4**

**Supplementary Tables S1-S4**

**Supplementary Materials and Methods**


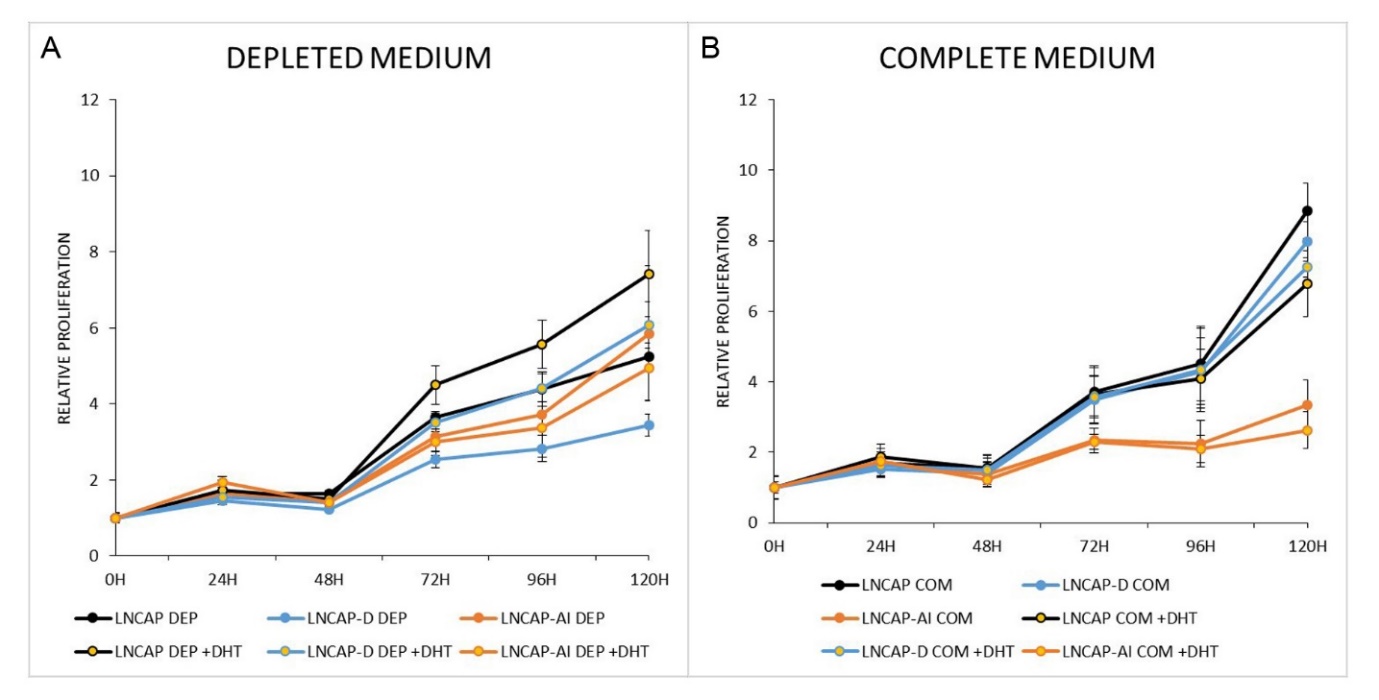


**Figure S1.** Growth curves of LNCaP, LNCaP-D and LNCaP-AI cells. (A) Growth curves in depleted medium. Lines with full dots represent growth curves without the addition of exogenous DHT whereas lines with yellow dots show cell growth with the addition of DHT. (B) Growth curves in complete medium. Lines with full dots represent growth curves without the addition of exogenous DHT whereas lines with yellow dots show cell growth with the addition of DHT.





**Figure S2.** Involvement of SHH in LNCaP-AI cells. (A) 50 nM siSHH successfully downregulates *SHH* expression in LNCaP-AI cells. (B) *SHH* silencing downregulates AR target gene (*PSA* and *KLK2*) expression levels. HH-GLI target gene *PTCH1* is also downregulated, while *GLI1* expression is unchanged. (C) LNCaP-AI cells grown as spheroids show increased *SHH* gene expression. (D) *PSA* expression is significantly upregulated in spheroid compared to adherent LNCaP-AI cells, while *KLK2* gene expression shows the same trend. * indicates P < 0.05.


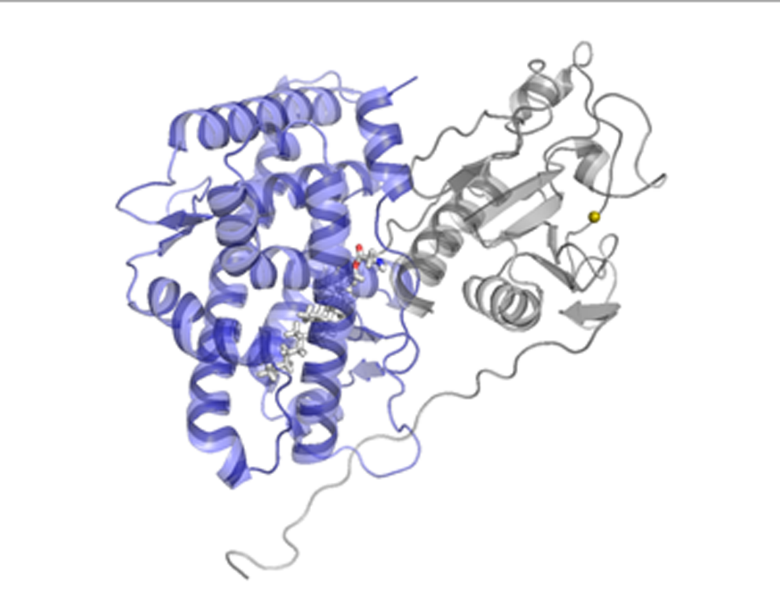


**Figure S3.** ARwt ̶ SHH-N ̶ cholesterol complex obtained by docking and used as initial structure for MD simulations. Proteins are represented as ribbons (AR – blue, SHH-N – gray) and cholesterol molecule is shown in stick presentation.

| 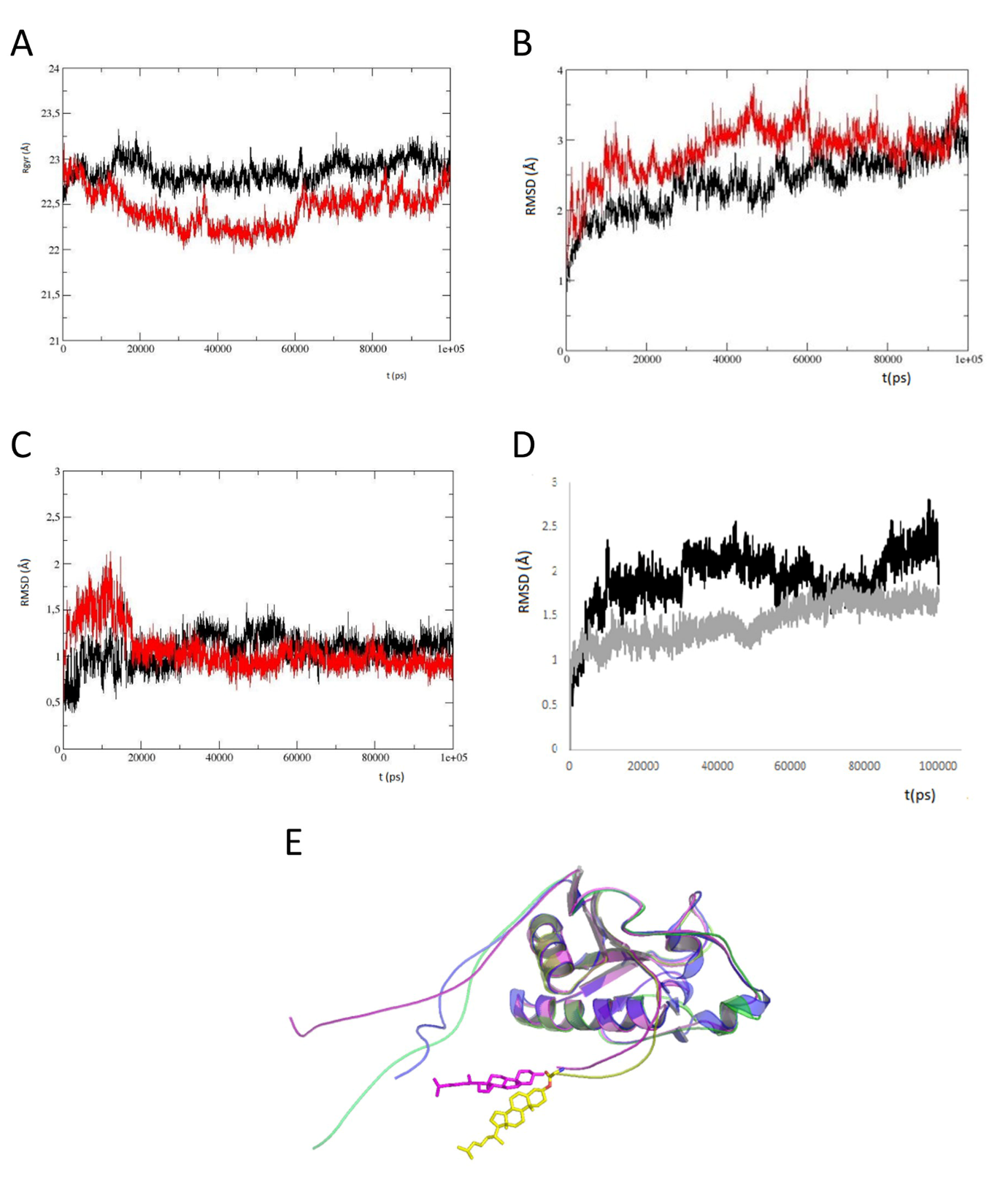 |  |
| --- | --- |
|  |  |

**Figure S4.** Geometrical analysis of the ARwt ̶ SHH-N ̶ cholesterol and T878A ̶ SHH-N ̶ cholesterol complexes during 100 ns of MD simulations for which the lowest binding free energy was determined. (A) Rgyr (Å). (B) RMSD (Å) (wt black line, and mutated red line). (C) RMSD (Å) of the cholesterol molecule during the specified simulations, D) RMSD (Å) of AR (gray) and SHH-N (black). (E) Overlay of the SHH structures: from the low energy MD simulation (green ribbon with cholesterol shown as yellow sticks), crystalographically determined structure of SHH (PDB code 3M1N3, blue) and the structure with the covalently bound cholesterol determined by cryoelectron microscope (PDB code 6RVD, magenta).

**Table S1.** Androgen-independent growth of LNCaP cells calculated as the growth for 5 days in depleted medium/growth in complete medium, without or with addition of exogenous DHT. Androgen independent cells have higher growth rates in androgen depleted medium which is not additionally increased by DHT.

| Cell line | Growth rate | Growth rate + DHT |
| --- | --- | --- |
| LNCaP | 0.59 | 1.10 |
| LNCaP-D | 0.43 | 0.84 |
| LNCaP-AI | 1.76 | 1.88 |

**Table S2.** MMGBSA energies and standard deviations (kcal/mol) calculated during 20 and 40 ns long segments of the 100 ns long MD trajectories of the ARwt ̶ SHH-N ̶ cholesterol (WT) and T878A ̶ SHH-N ̶ cholesterol complexes (MUT).

|  | ΔE_MM-GB_  0-20 ns | ΔE_MM-GB_  20-40 ns | ΔE_MM-GB_  40-80 ns | ΔE_MM-GB_  80-100 ns | <ΔE_MM-GB_>_100 ns_ (kcal/mol) |
| --- | --- | --- | --- | --- | --- |
| WT - 1 | -116 (±10) | -88 (±13) | -89 (±11) | -91 (±16) | -96 |
| WT - 2 | -80 (±9) | -79 (±11) | -89 (±10) | -89 (±10) | -84 |
| MUT - 1 | -85 (±10) | -88 (±16) | -79 (±11) | -87 (±10) | -84 |
| MUT - 2 | -88 (±12) | -80 (±13) | -68 (±12) | -81 (±12) | -79 |

**Table S3.** Population of the intermolecular hydrogen bonds (HB). Population of HB between residues *i* and *j*, belonging to AR (column) and SHH-N (row) respectively which appear in more than 30% of the sampled structures are given**.** The analysis was performed for the entire, 100 ns long trajectories for which the lowest average MMGBSA energy was determined during MD simulations of the ARwt ̶ SHH-N ̶ cholesterol (bold) and T878A ̶ SHH-N ̶ cholesterol complexes (underlined).

|  | ***AR*** | | | | | | | | | |
| --- | --- | --- | --- | --- | --- | --- | --- | --- | --- | --- |
| **SHH-N** | **N676** | **E679** | **G689** | **S704** | **E707** | **K718** | **R753** | **D768** | **S889** | **D891** |
| **G27** |  |  |  |  |  |  |  |  | **33**  34 |  |
| **R28** |  |  |  |  |  |  |  |  | **61** | **125**  133 |
| **R33** |  |  |  |  |  |  |  |  |  | **36**  115 |
| **R34** |  |  |  | **67** | **167** |  |  | **46** |  |  |
| **H35** |  |  | 48 |  |  |  |  |  |  |  |
| **P78** | **71** |  |  |  |  |  |  |  |  |  |
| **D83** |  |  |  |  |  | **84**  16 |  |  |  |  |
| **Q100** |  | **82**  16 |  |  |  |  |  |  |  |  |
| **K103** | **69** | **15**  62 |  |  |  |  |  |  |  |  |
| **K194** |  | 59 |  |  |  |  |  |  |  |  |
| **S195** |  |  |  |  |  |  | 37 |  |  |  |

**Table S4.** Primer sequences used for qPCR experiments

| **Gene** | **Forward** | **Reverse** | **Reference** |
| --- | --- | --- | --- |
| *RPLP0* | 5' GGCACCATTGAAATCCTGAGTGATGTG 3' | 5' TTGCGGACACCCTCCAGGAAGC 3' | Eichberger et al, 2004 [1] |
| *SHH* | 5' GAAAGCAGAGAACTCGGTGG 3' | 5' GGTAAGTGAGGAAGTCGCTG 3’ | Kallassy et al, 1997 [2] |
| *PTCH1* | 5' TCCTCGTGTGCGCTGTCTTCCTTC 3' | 5' CGTCAGAAAGGCCAAAGCAACGTGA 3' | Regl et al, 2002 [3] |
| *GLI1* | 5' CGGGGTCTCAAACTGCCCAGCTT 3' | 5' GGCTGGGTCACTGGCCCTC 3' | Chatel et al, 2007 [4] |
| *PSA* | 5′-AGTGCGAGAAGCATTCCCAAC-3 | 5′-CCAGCAAGATCACGCTTTTGTT-3′ | Li et al, 2018 [5] |
| *KLK2* | 5′-GCTGCCCATTGCCTAAAGAAG-3′ | 5′-TGGGAAGCTGTGGCTGACA-3′ | Li et al, 2018 [5] |

**Materials and Methods:**

Cell lines and Spheroid Culture

Human prostatic cell lines LNCaP and LNCaP-AI were cultured to obtain 3D spheroids. All three cell lines were cultured separately with each cell line being seeded on one lid of the respective tissue culture dish, 100 x 20 mm, by means of hanging drop system. Total number of drops per plate lid was 25 at 2 x 10^3^ cells per drop. Each tissue culture dish was supplemented with 5 ml of PBS (phosphate buffered saline) on the bottom, covered with the lid containing hanging drops. Cells were cultured for 72 hours at 37 °C, 95% relative humidity in a 5% CO_2_ and air atmosphere. To assess 3D spheroid formation, hanging drops were observed using BOECO Inverted Biological Microscope BIB-100.

Molecular Modeling

*Systems preparation and parametrization*

T877A homologue of AR was prepared in PyMOL by mutating Thr at position 877 to Ala. Cholesterol molecule was docked into AR ligand binding domain by the program AutoDock 4.2.6 [6]. Centre of the docking box of approximately 3·10^3^ Å^3^ was placed approximately in the middle of the ligand binding site, and the grid spacing was 0.375 Å. Ligands were considered flexible, and the docking was performed by Lamarckian Genetic Algorithm (LGA) using default parameters.

The AR-SHH-cholesterol and T877A-SHH-cholesterol complexes were built in PyMOL in a way that the cholesterol bound to the N-terminal domain of SHH was aligned with the lowest energy pose obtained by docking (Figure S1). The complexes were solvated in an octahedron box filled with TIP3P water molecules ensuring an 11 Å thick water molecules buffer around the protein. Cl- ions were added to neutralize the system.

*Molecular Dynamics (MD) Simulations*

Prior to molecular dynamics simulations, the protein geometry was optimized in three cycles with different constraints. In the first cycle (1500 steps), only water molecules were relaxed, while the protein and zinc atoms were restrained by the harmonic potential with a force constant of 32 kcal mol/Å2. In the second (2500 steps) cycle, the force constant of 12 kcal mol/Å^2^ was applied to the backbone while in the third cycle (4500 steps), all atoms were free to move. Followed heating, density equilibration and productive MD simulation. During heating from 0 to 300 K, the NVT ensemble was used, while all of the following simulations, density equilibration and productive MD simulations, were performed using NPT ensemble. The temperature (300 K) was held constant using Langevin dynamics with a collision frequency of 1 ps-1. Pressure was regulated by a Berendsen barostat. Time step was 1 fs during equilibration and 2 fs during productive MD simulations, respectively. Bonds involving hydrogen atoms were constrained using the SHAKE algorithm. For each set of data two set of 100 ns long simulations were performed with the GPU version of the pmemd (pmemd.cuda.MPI) program from the Amber software suite version 16 (https://ambermd.org/).

*Data analysis*

Data analysis (RMSD, RMSF calculations and H-bond analysis) was performed with the AmberTools16 module CPPTRAJ. The cutoff distance and angle for hydrogen bonds was set to default values of 3.0 Å and 135°, respectively. These relatively tight criteria ensured that only the most relevant interactions were taken into account. The hydrogen bonds population between residues (i and j) is calculated as the ratio of the number of trajectory frames containing hydrogen bond and the total number of frames. (HB_i,j_^pop^=N(frames with HB_i,j_)/N(frames total)). In the case of residues forming multiple hydrogen bonds, a sum of these values is given, which allows values larger than 100%. For example, if a glutamate forms hydrogen bonds with both carboxyl oxygens at the same time, the sum of hydrogen bonds might be above 100%. Such approach enabled better quantification of an amino acid residue importance in a complex stabilization while keeping the table dimensions within reasonable boundaries.

**References**

[1] T. Eichberger, G. Regl, M.S. Ikram, G.W. Neill, M.P. Philpott, F. Aberger, A.-M. Frischauf, FOXE1, a new transcriptional target of GLI2 is expressed in human epidermis and basal cell carcinoma, J. Invest. Dermatol. 122 (2004) 1180–1187. https://doi.org/10.1111/j.0022-202X.2004.22505.x.

[2] M. Kallassy, R. Toftgaard, M. Ueda, K. Nakazawa, I. Vŏrechovskỳ, H. Yamasaki, H. Nakazawa, Patched (ptch)-associated preferential expression of Smoothened (smoh) in human basal cell carcinoma of the skin, Cancer Research. 57 (1997) 4731–4735.

[3] G. Regl, G.W. Neill, T. Eichberger, M. Kasper, M.S. Ikram, J. Koller, H. Hintner, A.G. Quinn, A.-M. Frischauf, F. Aberger, Human GLI2 and GLI1 are part of a positive feedback mechanism in Basal Cell Carcinoma, Oncogene. 21 (2002) 5529–5539. https://doi.org/10.1038/sj.onc.1205748.

[4] G. Chatel, C. Ganeff, N. Boussif, L. Delacroix, A. Briquet, G. Nolens, R. Winkler, Hedgehog signaling pathway is inactive in colorectal cancer cell lines, International Journal of Cancer. 121 (2007) 2622–2627.

[5] N. Li, S. Truong, M. Nouri, J. Moore, N. Al Nakouzi, A.A. Lubik, R. Buttyan, Non-canonical activation of hedgehog in prostate cancer cells mediated by the interaction of transcriptionally active androgen receptor proteins with Gli3, Oncogene. 37 (2018) 2313–2325. https://doi.org/10.1038/s41388-017-0098-7.

[6] G.M. Morris, R. Huey, W. Lindstrom, M.F. Sanner, R.K. Belew, D.S. Goodsell, A.J. Olson, AutoDock4 and AutoDockTools4: Automated docking with selective receptor flexibility, J Comput Chem. 30 (2009) 2785–2791. https://doi.org/10.1002/jcc.21256.

**ORIGINAL UNPROCESSED BLOTS:**

**Figure 1 / β-ACTIN**


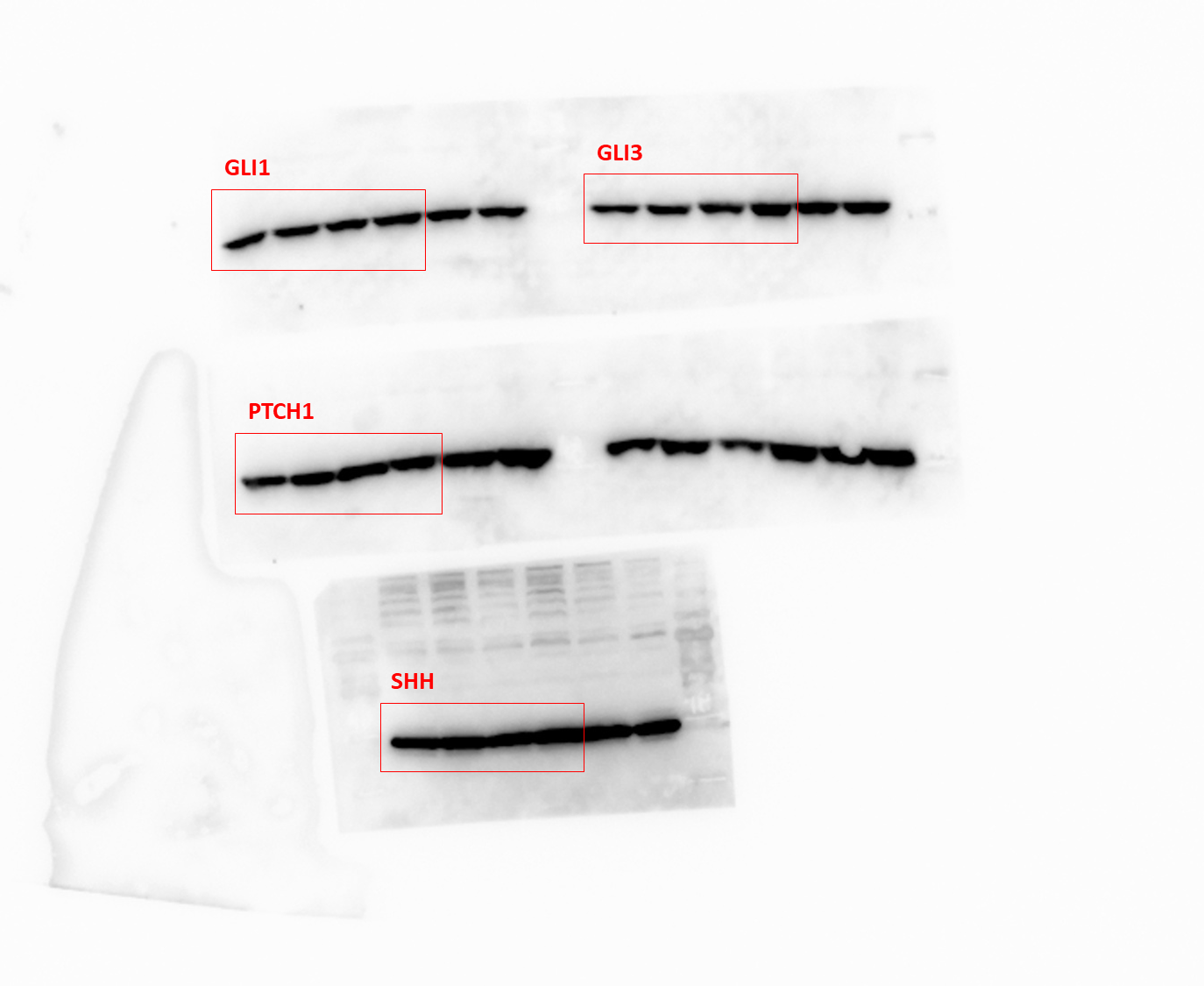


**Figure 1 / GLI1**


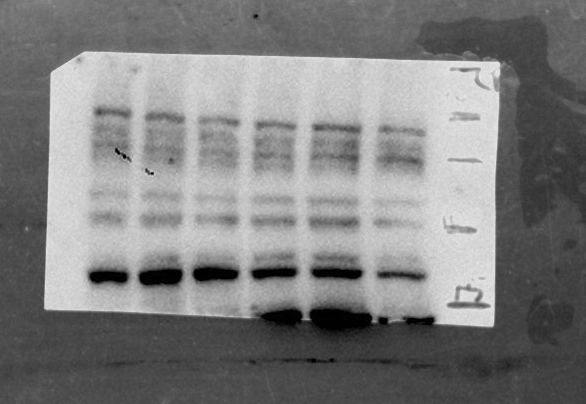


**Figure 1 / GLI3**


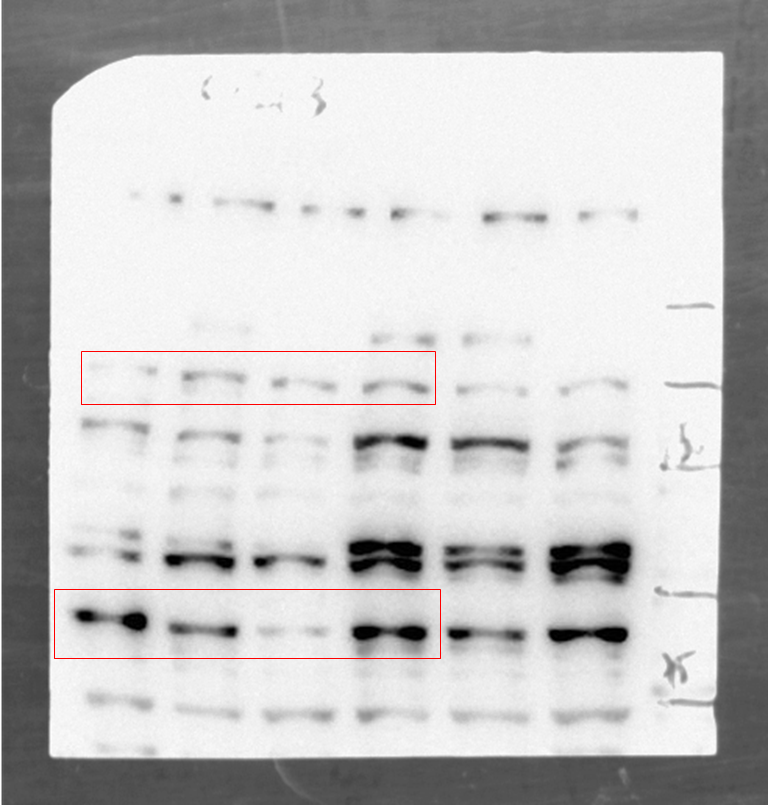


**Figure 1 / PTCH1**


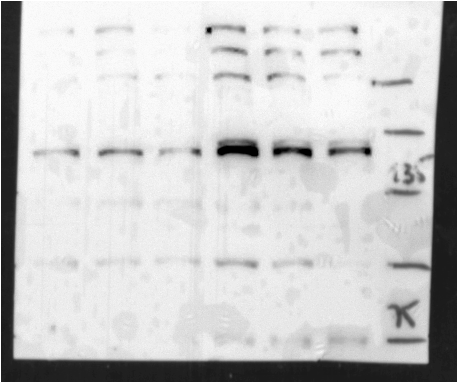


**Figure 1 / SHH**


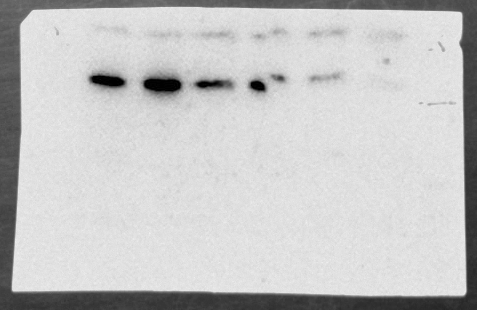


**Figure 2 / pSRC**


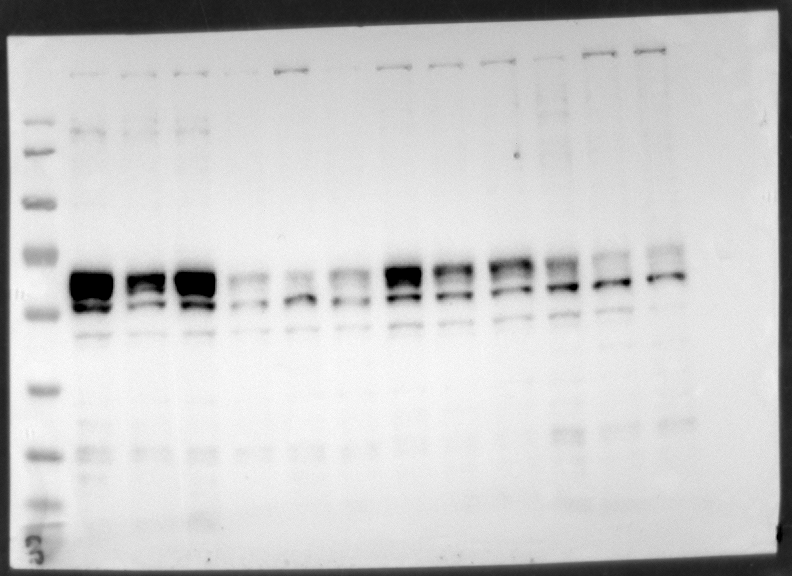


**Figure 2 / SRC**


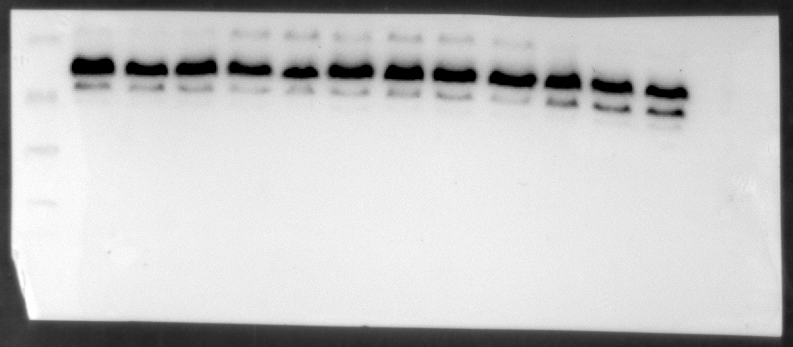


**Figure 3 / IP: SHH+AR**


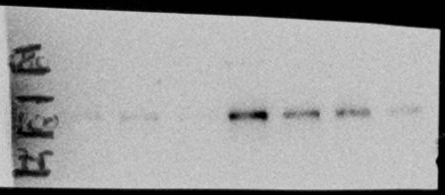


**Figure 3 / input AR**


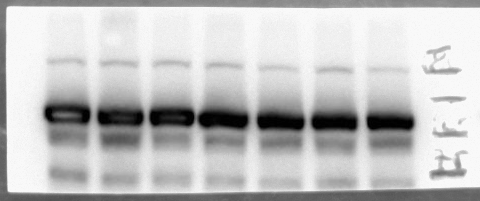

Supplement: Supplementary file 1 — Supplementary Information. [file 41598_2021_93971_MOESM1_ESM.docx]
